# Cost analysis of early discharge using combined copeptin/cardiac troponin testing versus serial cardiac troponin testing in patients with suspected acute coronary syndrome

**DOI:** 10.1371/journal.pone.0202133

**Published:** 2018-08-23

**Authors:** Thomas Reinhold, Evangelos Giannitsis, Martin Möckel, Lutz Frankenstein, Mehrshad Vafaie, Jörn O. Vollert, Anna Slagman

**Affiliations:** 1 Institute for Social Medicine, Epidemiology and Health Economics, Charité –Universitätsmedizin Berlin, Berlin, Germany; 2 Department of Angiology, Cardiology and Pneumology, University Hospital Heidelberg, Heidelberg, Germany; 3 Division of Emergency Medicine and CPU, Department of Cardiology, Charité –Universitätsmedizin Berlin, Berlin, Germany; 4 James Cook University (JCU), Cairns, Australia; 5 ThermoScientific-BRAHMS GmbH, Hennigsdorf, Germany; Ottawa Hospital Research Institute, CANADA

## Abstract

**Background:**

Symptoms indicating acute coronary syndrome are commonly seen in emergency rooms, but only 10% of patients are actually diagnosed with acute myocardial infarction (AMI). The Guidelines for the diagnosis of patients with suspected AMI include either multiple testing of cardiac troponin (cTN) or a single combined test of cTN and copeptin, which facilitates earlier diagnosis or exclusion of AMI. The aim of the present analysis was to investigate the impact of combined copeptin/cTN testing on health care resource consumption and related costs both during and after initial hospital treatment.

**Methods and results:**

The analysis was based on the BIC-8 trial and financial data of participating study sites. A cost analysis was carried out primarily from the hospital perspective and secondarily from the perspective of German statutory health insurers. The underlying assumptions of the investigation were tested for robustness in additional sensitivity analyses. In total, the data of 713 patients (n = 359 combined copeptin/cTN testing, n = 354 serial cTN testing) were evaluated. From a hospital perspective, the combined copeptin/cTN testing showed a reduced number of medical procedures and a lower frequency of inpatient admissions. The average staff time was significantly reduced by a mean of 49 minutes (95% confidence interval (CI) 46 to 53) per patient, accompanied by a significant mean reduction of 131 minutes (95%CI 104 to 158) in the time patients stayed in the emergency room. The initial hospital treatment was less cost-intensive. Over the entire study period, no significant cost differences were observed between the groups for health insurance.

**Conclusion:**

The combined copeptin/cTN testing has the potential to save costs and staff time in acute care and for the entire hospital stay. The primary explanations for these findings are early identification and ruling out patients without AMI along with the associated reduced need for acute medical treatment.

**Trial registration:**

ClinicalTrials.gov NCT01498731

## Introduction

Signs and symptoms suggestive of acute coronary syndrome (ACS) are commonly found in emergency departments (ED) and chest pain units (CPUs) but only 10% of these patients are actually diagnosed with acute myocardial infarction (AMI) [1]. The current guidelines for the management of patients with suspected non-ST-elevated myocardial infarction recommends using either serial cardiac troponin (cTn) testing or the combined testing of copeptin and cTn at admission to rule out AMI early [2]. The copeptin-based protocol has already been tested in the prospective randomized clinical BIC-8 trial (Biomarkers In Cardiology-8) [3]. The primary result of the BIC-8 trial showed the noninferiority of an early rule-out based on the proposed copeptin algorithm compared to standard of care with serial cTn testing regarding patient safety (30-day major adverse cardiac events (MACE)): copeptin 5.19% (95%CI 3.32 to 7.69), vs. standard 5.17% (95%CI 3.30 to 7.65)). In this trial MACE was defined as all-cause death or survived sudden cardiac death, AMI, rehospitalization for ACS, acute unplanned percutaneous coronary intervention (PCI), coronary artery bypass grafting (CABG), and documented life-threatening arrhythmias (ventricular tachycardia, ventricular fibrillation, complete atrioventricular block, including events during the index hospital stay) [3]. Additionally, in the experimental group, patient ED/CPU length of stay (LOS) was significantly reduced, and less diagnostic measures were necessary.

Thus, the objective of the present analysis was to investigate how much the use of combined copeptin/cTn testing affected resource consumption and related costs during the initial treatment of participants of the BIC-8 trial in an acute care setting as well as after hospitalization.

## Methods

The present health economic secondary analysis was based on BIC-8 trial data in combination with data requested from the financial departments of the involved study sites. However, the analysis was restricted to patients with hospital admission in only two university study sites in Germany (Berlin, Heidelberg). This restriction was determined for due to two reasons. First, comparisons of health economic results between different national health care systems are limited [4], so the analysis was restricted to patients from German study sites. In total, Berlin and Heidelberg recruited 713 patients. These patients were 79% of all BIC-8 patients (n = 902), so the restriction to these sites did not lead to substantial limitations regarding the included number of patients.

### Study design

In the BIC-8 trial (ClinicalTrials.gov registration number NCT01498731), patients with suspected ACS were included after they had contact to the ED in one of the participating study sites. Patients were randomized to either the copeptin group or the standard treatment group. In the standard group, patients were managed according to the current European Society of Cardiology (ESC) guidelines, including serial measuring of cTn. Copeptin values were also measured in the standard group but not revealed to the treating staff to assure standard care. In the copeptin group, cTn and copeptin were assessed, but in contrast to the standard group, the copeptin value was used for further patient management. In cases of negative results for both biomarkers, patients were considered to be low risk and could be early discharged into outpatient care. Before discharge, patients had a final visit to ensure their well-being. The final discharge decision was at the discretion of the attending physician who was allowed to overrule the biomarker result. All discharged patients had an outpatient cardiology appointment within a maximum of 3 days after discharge. In case of a positive copeptin result, patients were managed equal to the standard group. Copeptin was measured from the routine blood sample at admission using the Thermo Scientific B·R·A·H·M·S Copeptin ultrasensitive KRYPTOR assay. cTn was tested as by routine practice at the individual sites.

**Fig 1:**
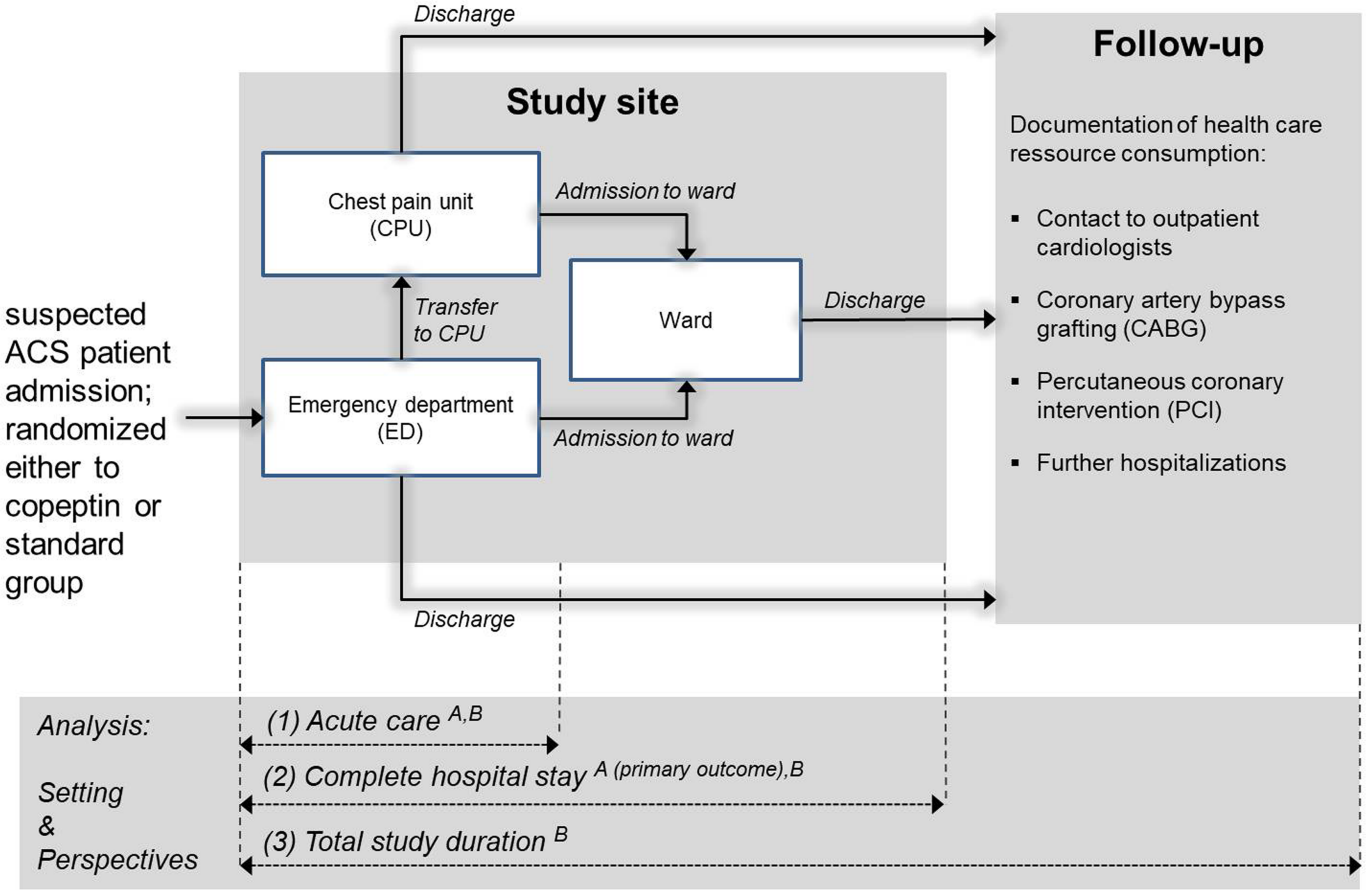
Study overview (including patient pathways through the study, definition of analysis setting [1 –acute care setting, 2 –complete initial hospital setting, 3 –Total study duration] and costing perspectives [A–hospital perspective, B–Statutory health insurance perspective]).

Possible pathways of the participating patients are shown in Fig 1. More details of the underlying study design and inclusion/ exclusion criteria are reported elsewhere [3].

### Types of economic analyses, analyzed time frame and costing perspectives

The economic analyses were focused on resource consumption and costs. This focus was selected because the BIC-8 trial has shown comparability between the groups in terms of MACE, which was the primary study outcome of this clinical trial [3]. This comparability was also given in the analyzed subgroup of patients from German study sites, where the proportion of MACE during the study was 5.01% (n = 18) in the copeptin group and 5.08% (n = 18) in the standard group.

In the present analysis different time frames were analyzed (Fig 1). Because we were interested in obtaining a picture of the ED/CPU processes, the analysis was completed in three steps: (1) the acute care setting, (2) the complete initial hospital setting, and (3) the 90 days follow-up period. Additionally, the analysis was performed from two different perspectives. Primary costs were analyzed from the perspective of the participating study center (setting 1: ED/CPU; setting 2: complete initial hospital stay). A secondary analysis was performed from the perspective of German statutory health insurers (setting 1: ED/CPU, setting 2: complete initial hospital stay; setting 3: 90-day follow-up).

### Outcomes

According to the time frame of the study and the costing perspectives, study outcomes were cost per patient in acute care setting of the ED/CPU (from both perspectives), cost per patient for the complete initial hospital setting (from both perspectives), and costs for the total study period (only statutory health insurance perspective). The predefined primary outcome was mean cost per patient for the complete initial hospital setting from the hospital perspective.

Furthermore we analyzed the number of patients receiving procedures, invested staff person-time and LOS in an acute care setting.

### Resource consumption

During the initial stay in the acute care setting of the ED/CPU, resource consumption was documented by participating sites in electronic case report forms (eCRFs). These forms included data the on number of blood samplings, electrocardiograms (ECG), echocardiography, medication, patient transfers and discharge. Additionally, to measure and report the person-time for medical staff in ED/CPU, the mean time (in minutes for medical doctors and nursing staff) needed for completing these single procedures were assumed based on expert hearing and summarized on a patient level. For patients transferred to the ward afterwards, no further information on concrete resource consumption in each ward was available.

After the initial hospital stay, patients had two follow-ups: 30 days and 90 days after randomization. For this period, the number of PCIs and CABGs, further hospitalizations, and clinical MACE was collected. These data were reported directly by the participating patients or their close relatives. According to the study protocol, all patients allocated to the copeptin group with early discharge from the ED/CPU should have had an outpatient cardiology appointment. Therefore, one outpatient cardiologist contact was assumed during the follow-up for each of these patients. For patients of the standard group who were directly discharged from ED/CPU, one outpatient contact to cardiologists was assumed for 50% of them.

### Cost assessment

The method for measuring the associated costs per patient depends on the costing perspective.

For the primary hospital perspective, the calculation of costs in the ED/CPU for all patients was based on the number of documented procedures during the study multiplied by the standardized unit costs. For each procedure, common national unit costs published in the DKG-NT handbook [5] (tariffs of the German hospital society) were used. The costs for medication were calculated based on the mean costs of daily defined dosages [6]. Because the KRYPTOR assay was provided based on a reagent rental contract, costs for copeptin testing were assumed to be 16 Euro per test according to Domenico at al. 2013 [7]. For patients transferred to the ward after their initial visit in the ED/CPU, the costs from the hospital perspective were provided by the financial departments of the participating centers. From the hospital perspective only the initial hospital stay was relevant, and resource consumption after and outside of this initial stay (during follow-up) was not considered.

The costs arising in the ED/CPU from the health insurers’ perspective were only available for patients without an inpatient stay. For these patients, we used the insurance outpatient reimbursement rates received and provided by the financial departments of the hospitals. Because patients transferred to the ward after initial ED/CPU visits are usually reimbursed by a DRG lump sum in Germany, a differentiation was not possible between ED/CPU costs and costs that occurred on the ward. However, for analyzing the complete hospital stay, these DRG costs were taken and combined with outpatient reimbursement rates for patients discharged from the ED/CPU. In contrast to the primary hospital perspective, resources and costs during the follow-up were cost-relevant for statutory health insurers. Therefore standard unit costs, reflecting the statutory health insurances perspective, were used to estimate the cost of outpatient cardiologist contacts [8–9], hospitalizations for CABG or PCI, and further inpatient stays during the follow-up period.

All underlying unit costs used in the present analyses are provided in Table 1.

**Table 1:** Unit costs used for monetization of medical resource consumption.

Ressource	Daily unit cost*	Source
Procedures in ED/CPU		
Physical examination	6.87 Euro	Based on Tariff of the German Hospital Society [5]
Electrocardiogram (ECG)	21.73 Euro	
Blood sampling	7.58 Euro	
Troponin testing	13.80 Euro	
Echocardiography	60.13 Euro	
Copeptin testing	16.00 Euro	Domenico et al. 2013 [7]
Medication in ED/CPU		
Nitro	0.28 Euro	Drug Regulation Report 2014 [6]
ASS	0.07 Euro	
Clopidogrel	0.52 Euro	
Prasugrel	2.77 Euro	
Ticagrelor	2.61 Euro	
Fondaparinux	5.88 Euro	
Unfractionated heparin	3.24 Euro	
Low molecular weight heparins	2.93 Euro	
GIIbIIIainhibitors	0.86 Euro	
Beta blockers i.v.	0.24 Euro	
Beta blockers oral	0.24 Euro	
Biuretics i.v.	0.16 Euro	
Biuretics oral	0.16 Euro	
Catecholamine’s	23.40 Euro	
Antibiotics	1.66 Euro	
Follow-up events		
Outpatient cardiologist visits	70.60 Euro	Own calculation: based on Krauth et al.2005 [8] and Bock et al.2015 [9]
Hospitalization for PCI	3619.75 Euro	Own calculation: Case Mix index cardiology 2012 (1.21) multiplied with German DRG base rate in 2012 (2991.53 Euro)
Hospitalization for CABG	12609.29 Euro	Own calculation: CABG DRG F06F (cost weight 4.215) multiplied with German DRG base rate in 2012 (2991.53 Euro)
Further hospitalization	2991.53 Euro	DRG base rate in 2012 (2991.53 Euro)

*values were varied within a range of ±50% maximum in sensitivity analysis

### Statistical analysis

Data analysis was based on the intention to treat (ITT) approach. For analyzing the results for the complete study period from a statutory health insurance perspective, only patients with complete follow-up information were included. No missing values were replaced.

Baseline characteristics were presented as proportions (%) or mean values with standard deviations (SD). The results of LOS and person-time invested by medical staff were tested by using generalized linear models for significant differences. To consider the skewed nature of cost data and potential differences between study arms, a generalized linear model with gamma distribution and log link function was used for results of cumulated costs. Adjusted results are shown as expected means and 95% confidence intervals (95%CIs) and mean group differences including 95%CI. The results were adjusted for gender and age; individual risk factors including diabetes, hypertension, hyperlipidemia, family history of myocardial infarction, smoking status; and medical history such as known coronary artery disease, prior myocardial infarction, chronic heart failure, primary valve disease, cardiomyopathy, prior percutaneous coronary intervention and prior coronary artery bypass graft. For the statistical model, default settings of the statistical software were used. Thus the value for continuous covariates was set to its overall mean estimate. In our model, the only continuous covariate was “age”, assuming an equal mean age of 53.19 years for both groups. All other covariates used in the analysis had dichotomous characteristics (yes/no) and were assumed with a share of 50% each in the groups. Single cost data additionally were presented as raw means and 95% confidence intervals as well as median values including interquartile range (75th and 25th percentiles).

The significance level was defined at 5% (two-sided). Because the primary outcome was defined *a priori*, no adjustment for multiple testing was conducted [10]. Therefore, significant results for secondary outcomes should be seen as hypothesis generating, not as definitive findings. For all statistical analyses, SPSS version 23 was used. All results were double checked by a second statistician using SAS 9.4.

### Sensitivity analysis

In economic evaluations of health interventions, the sensitivities of a cost result to changes in modeling assumptions, data variation, and sampling error are important. Any uncertainties in our analysis would result essentially from two factors: the assumption of the calculation (standardized unit costs) and the heterogeneity of the patient sample. Therefore, uncertainty was tested in additional sensitivity analyses for the primary outcome (mean cost per patient from the hospital perspective for the complete initial hospital setting) in combination with person-time invested by medical staff in ED/CPU. A probabilistic sensitivity analysis was used to address the uncertainty resulting from assumptions on unit costs and time invested by staff. Therefore, a Monte Carlo simulation process [11] was conducted that involved running the analyses 1000 times using randomly sampled values for each of the standardized unit costs shown in Table 1 and varying assumptions on person-time for medical staff simultaneously. For varying cost inputs a gamma distribution, for data on person-time a normal distribution was assumed. This simulation was based on the predefined input data range of ±50% maximum. Additionally, a bootstrap analysis with random 1000-fold resampling of the original population was performed to determine to what extent the results may vary due to many replications of a trial. This analysis accounted for the heterogeneity around all health care resource consumption observed in the study.

Both results of the Monte-Carlo simulations and the bootstrap samples were plotted into the four-quadrant diagram, which gives graphical information on the results’ robustness. The sensitivity analyses were performed by using MS Excel 2010.

## Results

### Baseline characteristics

Data of 713 patients (copeptin group, n = 359; standard group, n = 354) were available including study documentation in electronic case report forms (eCRFs) and were complemented by data provided by the financial departments of the participating study sites. Regarding patient characteristics, both groups were well balanced in a number of the observed items (Table 2).

**Table 2:** Patients characteristics at baseline.

	Copeptin (n = 359)	Standard (n = 354)
Female patients (%)	40.4	35.6
Age (mean±SD)	52.9±16.2	53.3±15.4
Risk factors		
BMI (mean±SD)	27.2±5.0	27.4±4.6
Diabetes (%)	11.7	13.0
Hypertension (%)	55.2	55.9
Hyperlipidaemia (%)	38.2	44.4
Family history of MI (%)	29.5	24.3
Smoker (%)	32.0	33.1
Ex-smoker (%)	34.0	31.1
GRACE-score (mean±SD)	80.1±26.2	79.7±26.3
TIMI risk score (mean±SD)	1.7±1.2	1.7±1.1
Medical history		
Known CAD (%)	23.4	21.2
Prior MI(%)	10.9	12.4
CHF (%)	8.4	4.2
PVD (%)	7.5	8.2
Cardiomyopathy (%)	3.1	1.1
Prior PCI (%)	20.1	18.9
Prior CABG (%)	4.5	3.4

Abbreviations: SD–standard derivation; BMI–body mass index; MI–Myocardial infarction; CAD—coronary artery disease; CHF–chronic heart failure; PVD—primary valve disease; PCI—percutaneous coronary intervention; CABG—coronary artery bypass graft

The mean age of the included patients was 53 years, and most patients were men. Both groups had comparable distribution of cardiovascular risk factors such as increased body mass index (BMI), hypertension or smoking status. Potential relevant differences were detectable for hyperlipidemia, diabetes, family history of myocardial infarction and the proportion of ex-smokers. Some differences were also observed regarding to the medical history of the patients, especially for prior chronic heart failure and cardiomyopathy.

### LOS and staff person-time in acute care setting (ED/CPU)

**Fig 2:**
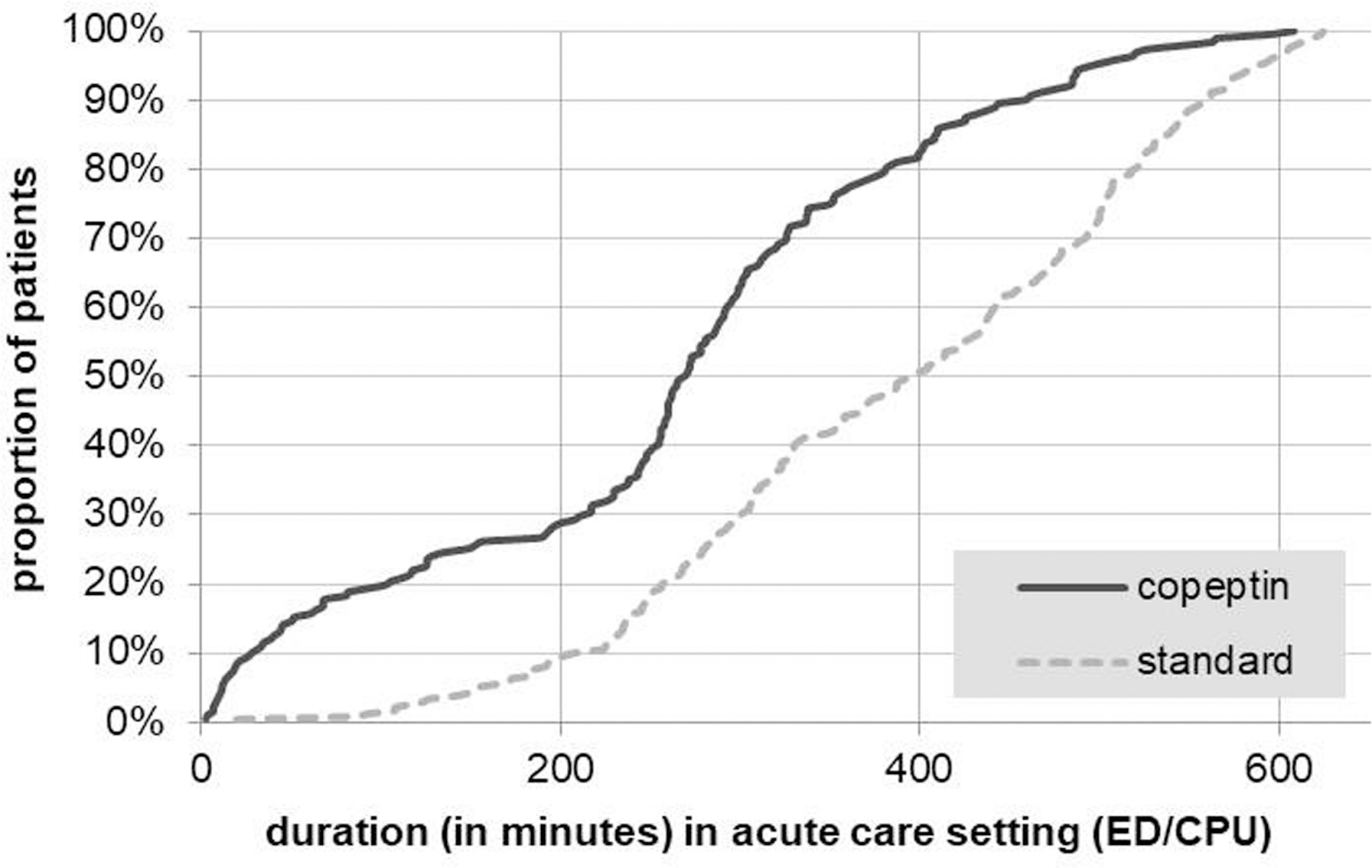
Patients’ length of stay (LOS) in the acute care setting of emergency department/Chest pain unit (ED/CPU).

Patients who received combined copeptin/cTn testing had a significant lower LOS in the ED/CPU than those who received standard care (copeptin: adjusted mean LOS 244 minutes (95%CI 181 to 307); standard: adjusted mean LOS 375 minutes (95%CI 309 to 440), adjusted mean difference -131 minutes (95%CI -158 to -104); p<0.001). This result was primarily explained by a lower number of patients receiving medical procedures (Table 3) and more patients discharged earlier to outpatient care or transferred to the ward (Fig 2).

**Table 3:** Cost per patient from hospital perspective by timeframe.

		Copeptin			Standard		
		n affected	mean (95%CI)	median (IQR)	n affected	mean (95%CI)	median (IQR)
(1) Acute care setting		n = 359			n = 354		
Phycical examination		359	6.87 Euro (6.87 to 6.87)	6.87 Euro (6.87; 6.87)	354	6.87 Euro (6.87 to 6.87)	6.87 Euro (6.87; 6.87)
1st ECG		359	21.73 Euro (21.73 to 21.73)	21.73 Euro (21.73; 21.73)	354	21.73 Euro (21.73 to 21.73)	21.73 Euro (21.73; 21.73)
2nd ECG		27	1.63 Euro (1.04 to 2.23)	0.00 Euro (0.00; 0.00)	173	10.62 Euro (9.48 to 11.76)	0.00 Euro (0.00; 21.73)
1st blood sampling		359	7.58 Euro (7.58 to 7.58)	7.58 Euro (7.58; 7.58)	354	7.58 Euro (7.58 to 7.58)	7.58 Euro (7.58; 7.58)
3 h blood sampling		44	0.93 Euro (0.67 to 1.19)	0.00 Euro (0.00; 0.00)	278	5.95 Euro (5.63 to 6.28)	7.58 Euro (7.58; 7.58)
6 h blood sampling		35	0.74 Euro (0.51 to 0.97)	0.00 Euro (0.00; 0.00)	202	4.33 Euro (3.93 to 4.72)	7.58 Euro (7.58; 7.58)
Troponin testing		359	16.83 Euro (16.06 to 17.61)	13.80 Euro (13.80; 13.80)	354	32.51 Euro (31.65 to 33.06)	27.60 Euro (27.60; 41.40)
Copeptin testing		359	16.00 Euro (16.00 to 16.00)	16.00 Euro (16.00; 16.00)	0	-	-
Echocardiography		19	3.18 Euro (1.78 to 4.58)	0.00 Euro (0.00; 0.00)	84	14.27 Euro (11.59 to 16.95)	0.00 Euro (0.00; 0.00)
Medication		36	1.76 Euro (0.53 to 2.98)	0.00 Euro (0.00; 0.00)	200	4.60 Euro (2.93 to 6.26)	0.62 Euro (0.00; 3.49)
Sum acute care setting		359	77.26 Euro (73.92 to 80.60)	65.98 Euro (65.98; 65.98)	354	108.46 Euro (104.69 to 112.22)	114.47 Euro (73.31; 118.47)
**Sum acute care setting** adjusted mean*	**p<0.001**	**359**	**81.17 Euro (72.46 to 90.92)**		**354**	**114.61 Euro (101.98 to 128.79)**	
(2) Complete hospital stay		n = 359			n = 354		
Sum acute care setting		359	76.99 Euro (73.76 to 80.22)	65.98 Euro (65.98; 65.98)	354	108.38 Euro (104.66 to 112.10)	114.47 Euro (73.31; 118.47)
Ward		97	683.79 Euro (324.37 to 1043.21)	0.00 Euro (0.00; 291.78)	219	582.71 Euro (452.24 to 713.18)	347.10 Euro (0.00; 492.03)
Sum complete hospital stay		359	761.05 Euro (400.91 to 1121.19)	65.98 Euro (65.98; 373.45)	354	691.16 Euro (558.46 to 823.87)	453.53 Euro (93.00; 606.24)
**Sum complete hospital stay (primary outcome)** adjusted mean*	**p = 0.025**	**359**	**473.50 Euro (280.64 to 798.89)**		**354**	**633.97 Euro (377.57 to 1064.47)**	

* adjusted for gender and age; individual risk factors: diabetes, hypertension, hyperlipidaemia, family history of myocardial infarction, smoking status; medical history: known coronary artery disease, prior myocardial infarction, chronic heart failure, primary valve disease, cardiomyopathy, prior percutaneous coronary intervention, prior coronary artery bypass graft

**Fig 3:**
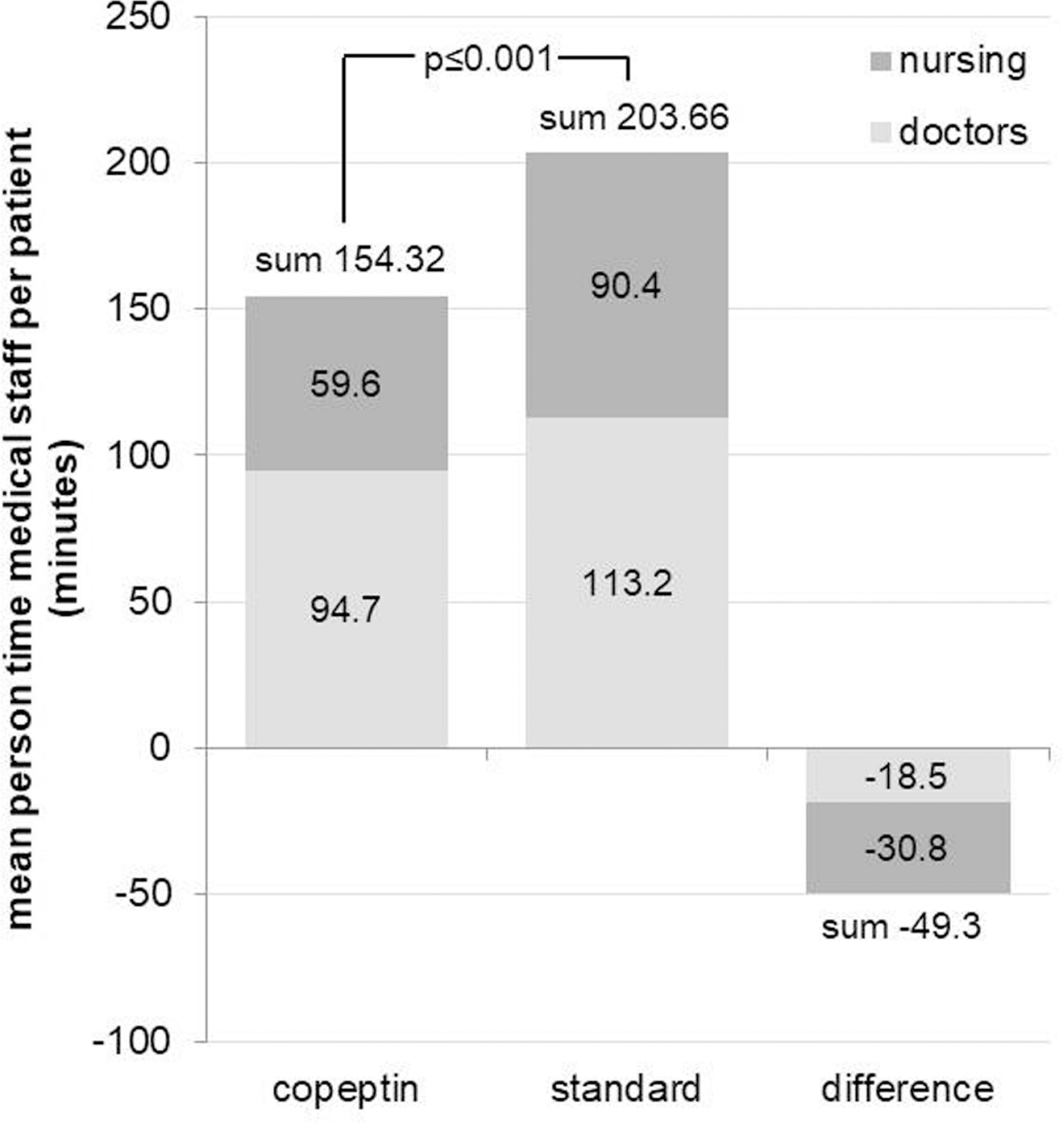
Adjusted mean person-time medical staff per patient in the acute care setting of emergency department/Chest pain unit (ED/CPU).

Particularly, the differences in the number of medical procedures had a direct effect on the time resources spent by the medical staff for treating each patient. The total mean person-time differed significantly (p<0.001) between the groups (Fig 3).

The adjusted total mean invested staff person-time for the copeptin group was approximately 154 minutes (95%CI 146 to 163) and 204 minutes (95%CI 195 to 212) for standard group, resulting in significant (p<0.001) mean difference of approximately -49 minutes (95% CI -53 to -46). Significant group differences were also detectable regarding the time invested for treatment by the type of professional groups. The mean staff time saved in the copeptin group was 31 minutes (95%CI 29 to 33, p<0.001) per patient for nursing staff and about 19 minutes (95%Ci 16 to 21, p<0.001) per patient for physicians.

### Costs per patient from primary hospital perspective

Due to the lower number of medical procedures, the copeptin group had significantly (p<0.001) lower total costs in the acute care setting of the ED/CPU from a hospital perspective (Table 3). The adjusted mean cost per patient was 81.17 Euro (95%CI 72.46 to 90.92) in the copeptin group and 114.61 Euro (95%CI 101.98 to 128.79) in the standard group (adjusted mean difference -33.42 Euro (95%CI -39.52 to -27.35)). These group differences can be explained in particular by a lower number of patients who received further blood samplings after 3 and 6 h, had echocardiograms, and took medications in the copeptin group (Table 3).

These results are also confirmed with respect to the total costs during the entire hospital stay (including the costs for inpatients with inpatient billing), which are defined as the primary outcome of this analysis. Thus, the mean total cost per patient was significantly lower for the copeptin group (copeptin: adjusted mean 473.50 Euro (95%CI 280.64 to 798.89); standard: adjusted mean 633.97 Euro (377.57 to 1064.47); adjusted mean difference -160.47 Euro (95%CI -301.21 to -19.73); p = 0.025).

### Costs per patient from secondary statutory health insurers’ perspective

From a statutory health insurers’ perspective (Table 4), patients in the copeptin group seem to be slightly more expensive during their stay in the acute care setting (copeptin: adjusted mean 82.70 Euro (95%CI 67.30 to 101.62); standard: adjusted mean 72.55 Euro (95%CI 58.20 to 90.43); adjusted mean difference 10.15 Euro (95%CI 3.48 to 16.82); p = 0.003).

**Table 4:** Cost per patient from statutory health insurers’ perspective by timeframe.

		Copeptin			Standard		
		n affected	mean (95%CI)	median (IQR)	n affected	mean (95%CI)	median (IQR)
(1) Acute care setting^#^		n = 262			n = 135		
Sum acute care setting		262	89.69 Euro (82.82 to 96.55)	77.10 Euro (66.15; 79.90)	135	77.47 Euro (68.13 to 86.82)	66.02 Euro (66.02; 66.02)
**Sum acute care setting** adjusted mean*	**p = 0.003**	**262**	**82.70 Euro (67.30 to 101.62)**		**135**	**72.55 Euro (58.20 to 90.43)**	
(2) Complete hospital stay		n = 359			n = 354		
Sum complete hospital stay		359	857.29 Euro (484.02 to 1230.56)	77.10 Euro (76.46; 589.02)	354	758.20 Euro (617.70 to 898.70)	611.19 Euro (66.02; 654.08)
**Sum complete hospital stay** adjusted mean*	**p = 0.078**	**359**	**655.06 Euro (388.79 to 1103.67)**		**354**	**805.40 Euro (481.80 to 1346.34)**	
(3) Total study duration^†^		n = 321			n = 312		
Sum complete hospital stay		321	908.73 Euro (492.56 to 1324.90)	77.10 Euro (76.46; 600.10)	312	730.96 Euro (581.94 to 879.97)	610.98 Euro (66.02; 654.08)
Outpatient cardiologist follow-up		234	51.47 Euro (48.01 to 54.92)	70.60 Euro (0.00; 70.60)	62	13.91 Euro (11.99 to 15.84)	0.00 Euro (0.00; 35.30)
Further hospitalization follow-up		29	354.14 Euro (214.89 to 493.39)	0.00 Euro (0.00; 0.00)	21	287.65 Euro (160.72 to 414.57)	0.00 Euro (0.00; 0.00)
Hospitalization for PCI follow-up		38	496.17 Euro (339.09 to 653.24)	0.00 Euro (0.00; 0.00))	26	324.85 Euro (200.69 to 449.01)	0.00 Euro (0.00; 0.00)
Hospitalization for CABG follow-up		3	117.84 Euro (0.00 to 251.28)	0.00 Euro (0.00; 0.00)	2	80.82 Euro (0.00 to 193.10)	0.00 Euro (0.00; 0.00)
Sum follow-up		321	1019.61 Euro (762.68 to 1276.54)	70.60 Euro (70.60; 70.60)	312	707.24 Euro (474.30 to 940.18)	0.00 Euro (0.00; 35.30)
Sum total study duration		321	1928.34 Euro (1352.97 to 2503.72)	156.21 Euro (147.70;2578.85)	312	1438.20 Euro (1156.35 to 1720.04)	620.98 Euro (101.32;684.30)
**Sum total study duration** adjusted mean*	**p = 0.093**	**321**	**1610.08 Euro (918.32 to 2822.91)**		**312**	**1301.02 Euro (744.46 to 2273.69)**	

^#^ only available for patients with outpatient billing

^†^ only available for patients with complete follow up

* adjusted for gender and age; individual risk factors: diabetes, hypertension, hyperlipidaemia, family history of myocardial infarction, smoking status; medical history: known coronary artery disease, prior myocardial infarction, chronic heart failure, primary valve disease, cardiomyopathy, prior percutaneous coronary intervention, prior coronary artery bypass graft

However, the informative value of this result is limited because it was restricted to patients with outpatient billing and did not include all patients who were reimbursed for inpatient treatment by a DRG. For this reason, the costs of the entire hospital stay including all patients are more meaningful, and the principal findings from the hospital perspective are essentially confirmed here. Thus, for health insurers, the complete hospital stay of the copeptin group was associated with adjusted mean costs per patient of 655.06 Euro (95%CI 388.78 to 1103.67) compared to 805.40 Euro (95%CI 481.80 to 1346.34) in the standard group (adjusted mean difference -150.34 Euro (95%CI -317.61 to 16.93 to); p = 0.078), primarily explained by the group differences in inpatient billing.

During the follow-up period, these savings were partly compensated for by higher mean costs in the copeptin group. Because patients with early rule-out should have had an outpatient cardiologist’s visit, additional costs associated with this physician contact occurred in the copeptin group. Additionally, during the study follow-up hospitalization costs were higher in the copeptin group. Finally, the adjusted total mean costs during the complete study duration were not significantly higher for the copeptin group (copeptin: adjusted mean 1610.08 Euro (95%CI 918.32 to 2822.91); standard: adjusted mean 1301.02 Euro (95%CI 744.46 to 2273.69); adjusted mean difference 309.05 Euro (95%CI -51.72 to 669.83); p = 0.093).

### Sensitivity analysis

**Fig 4:**
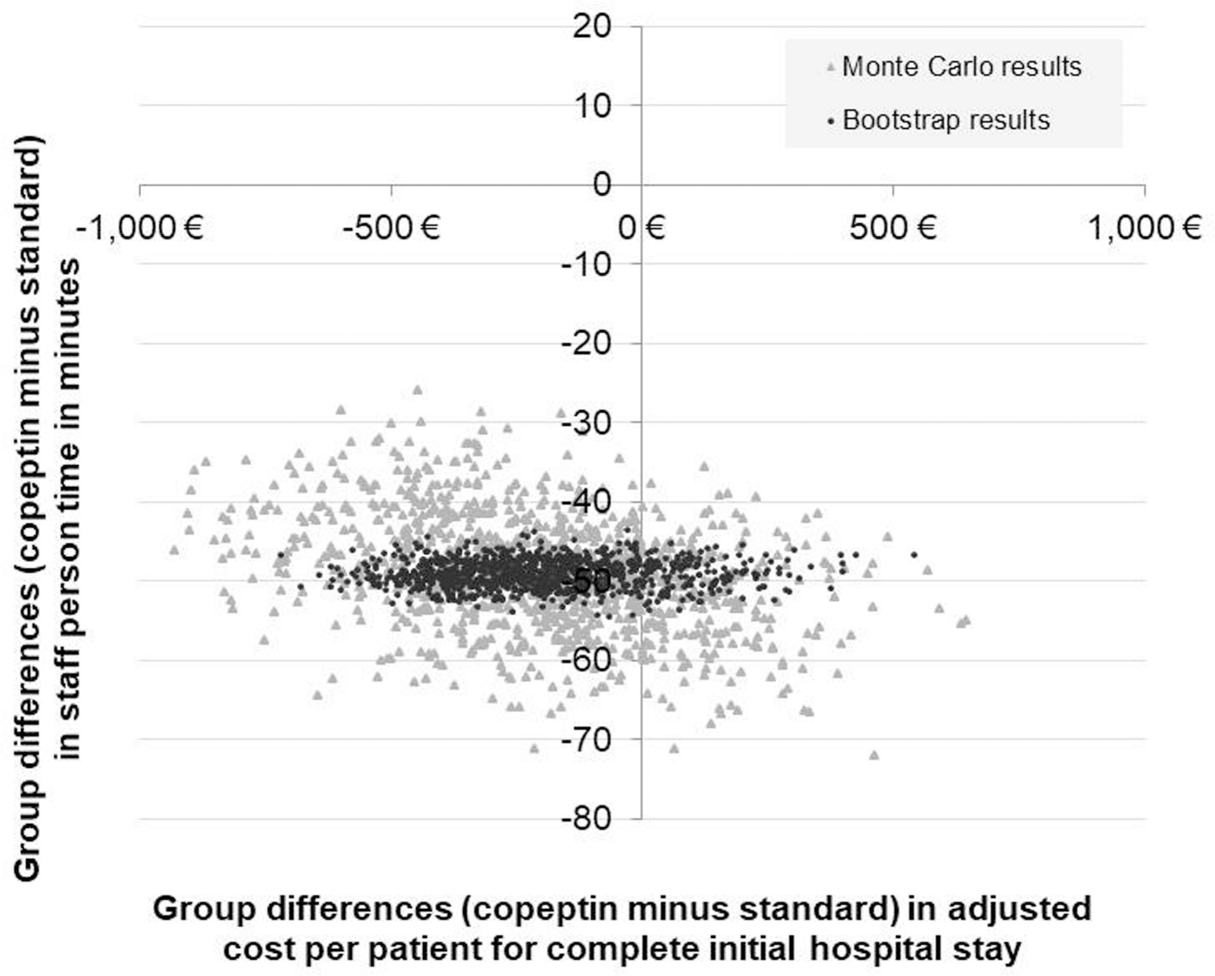
Sensitivity analyses of the primary outcome (adjusted mean cost per patient from hospital perspective for the complete initial hospital setting) in combination with person-time for medical staff in the acute care setting of emergency department/Chest pain unit (ED/CPU).

The simultaneous random sampling of unit cost assumptions and assumptions on person-time for medical staff, which was realized in the Monte Carlo analysis within a range of ±50%, essentially confirms the robustness of the primary outcome result. Both cost differences between the groups and person-time for medical staff were comparably stable with benefits for the copeptin group. The same applied to the results of the bootstrap analysis, which accounted for potential uncertainty around all health care resource consumption and potential outliers observed in the study participants. Fig 4 shows a scatter plot of all replicated results for both types of sensitivity analyses.

Most single dots are located in the lower left-hand quadrant, indicating a probability of 81% for lower costs and staff time savings for patients with copeptin testing. This means that the primary outcome is sufficiently confirmed even after considering alternative assumptions or patient distributions.

## Discussion

From an economic point of view, our analysis demonstrated the usefulness of copeptin testing as an additional marker for the early rule-out of patients with suspected ACS in emergency departments. From the hospital perspective, this testing saves person staff time and reduces LOS in the ED/CPU. Therefore, the use of this testing may be a helpful strategy to handle overcrowding in the acute care setting and to save hospital resources for the treatment of other severely ill patients. Additionally, the use of copeptin testing saves costs in treatment, especially by an early identification of patients without myocardial infarction requiring less medical acute care procedures. More efficient running hospitals can be seen as an added value to a health care system. From a statutory health insurers’ perspective, the combined copeptin/cTn testing was related to lower costs for initial hospital admission primarily due to a reduced number of patients in need of inpatient medical care. During the complete study period both groups were not significantly different in term of costs.

To our knowledge this is the first economic evaluation of copeptin for early rule-out of ACS in an emergency department or CPU setting in Germany. Our principal findings are in line with the results of a previous study from Italy that demonstrated the association of copeptin testing with cost savings and less procedural efforts [7]. Our findings are also confirmed by other studies that compared the economic consequences of accelerated rule-out protocols in ACS patients with repeated measurements. Ambavane et al. 2017 compared a 1h rule-out algorithm based on high sensitivity troponin T (hsTnT) with standard of care treatment in a nonrandomized, multicenter design [12]. Thus, in this study, all patients were diagnosed with repeated measurements, and cost-savings and reduction of LOS and staff time were only assumption-based findings. The investigators estimated an ED-LOS of 4.3h for early rule-out treatment and 6.5h for the standard treatment. These estimations were confirmed in our study with a randomized approach. Additionally, the total in-hospital costs were found to be significantly lower for early rule-out treatment than for standard of care treatment. Another study by Cheng et al. 2016 investigated an accelerated hsTnT diagnostic protocol (Brisbane protocol) in a tertiary care hospital in Australia using a pre-post comparison [13]. This study also showed a reduction in expected LOS and costs. Even though their standard group had a shorter LOS in the ED (median 5.6h) than our standard group, the reduction in ED LOS was lower when the Brisbane protocol was applied (median 4.7h). The Brisbane protocol includes a second hsTn testing 2h after admission, which may explain this lower reduction in ED LOS. In line with the findings of our study, overall in-hospital costs were lower when the accelerated rule-out was applied; however, ED costs were higher for the Brisbane protocol group than the standard treatment group (standard AUD 882; Brisbane protocol AUD 976). The study group from Brisbane also published a cost prediction model and an economic microsimulation applied in an observational cohort [14]. In this simulation, five different hsTn-based algorithms were compared, and the results again showed that early rule-out algorithms reduce hospital costs and LOS in the ED. These effects were primarily seen for non-ACS patients; there were no differences regarding these outcome variables in ACS patients. In a randomized approach, Kaambwa et al. 2017 further compared conventional cardiac troponin testing to hsTn and demonstrated that hsTnT leads to fewer adverse events but also higher costs during a 12-month follow-up period [15]. The authors concluded that substantial changes in clinical practice are required in order to reach cost-effectiveness of hsTnT as a routine procedure. Although the studies are less comparable, this finding was in contrast to our analysis. Even though the BIC-8 trial had a shorter follow-up period we found no significant differences regarding health care costs between the groups when mean values were analyzed over the complete study duration. Additionally, Vaidya et al. 2014 compared hsTnT to conventional troponin testing based on a decision analytical model. They concluded that hsTnT is a cost effective diagnostic alternative to conventional cTnT [16]. Another study comparing diagnostic protocols was published by Shortt et al. in 2017 [17]. They investigated the cost-effectiveness of diagnostic protocols based on troponin in combination with glycemic biomarkers (glucose, HbA1c) compared to the guideline recommended 0h algorithm using an observational study design. The authors concluded that the application of hsTnT in combination with glucose at presentation is more safe and cost-effective than the 0h hsTnT rule-out algorithm.

The present analysis was particularly characterized by its randomized design and comparatively high number of participants. Most of the other economic evaluations mentioned above, however, did not apply randomized designs. Furthermore, in the present analysis, it was possible to combine different data sources in the analysis, including real cost information provided by the participating hospitals. Nevertheless, the analysis contains potential limitations that readers should take into account when interpreting the results. First, the results were based on a number of different assumptions, such as the person minutes spent for medical procedures in acute care and the unit costs used for monetization of resources. To evaluate associated uncertainties, we conducted sensitivity analyses allowing varying underlying assumptions within realistic ranges, and these analyses confirmed our major findings. Second, this study had some statistical outliers, and as a common difficulty when analyzing cost data, we had to decide how to handle them. In our analysis five patients (copeptin n = 4, standard n = 1) had costs of more than 10000 Euro during their inpatient stay. In addition to their cardiac complaints, these patients had other accompanying severe diagnoses (e.g. ICD B90.9 sequelae of respiratory and unspecified tuberculosis, ICD N.17 acute renal failure, ICD J44 chronic obstructive pulmonary disease), which probably explained their high costs. Nevertheless, in our analyses, we decided to involve all patients in order to reflect real world and to avoid subjective inclusion/exclusion decisions. Third, our analysis used study sites that were located in an urban university setting, which restricts the generalization of the results (e.g. for smaller hospitals in rural areas). Nevertheless, BIC-8 was a multicenter study, and two study centers were considered in this analysis. Compared to other cost-effectiveness studies that were primarily based on single-center data or assumption-based model calculations, the use of real BIC-8 data in our analyses was an improvement. However, the transferability of our findings (especially for cost results) to other national health care systems is limited, as national health systems differ structurally in many aspects by country [4]. Fourth, the present economic evaluation was performed in a retrospective setting, because the underlying BIC-8 trial was initially designed as a clinical study and did not consider planning for health economic questions. For this reason, not all possible cost-relevant outcomes were taken into account, especially in the follow-up of the analysis. Fifth, the follow-up period of our analysis was not longer than 90 days, which may be too short to reflect all relevant long-term consequences. A comprehensive health economic analysis would require the consideration of all relevant resources and costs (e.g. medication after discharge) over a sufficiently long-time period, a condition that could not be fulfilled using the retrospective study setting. Further studies investigating the use of biomarkers should include possible health economic analyses during the study planning phase.

## Conclusion

This is the first extensive evaluation of cost and resource consumption associated with the application of a single combined copeptin/cTn early rule-out protocol in patients with suspected ACS in the ED in Germany. Based on our findings, a management process integrating early rule-out testing with combined copeptin/cTn in these patients is not only as safe as serial cTn testing but also bears the potential to save staff time and costs accumulated in the acute care setting and the entire hospital stay.
